# The FKBP4 Gene, Encoding a Regulator of the Androgen Receptor Signaling Pathway, Is a Novel Candidate Gene for Androgen Insensitivity Syndrome

**DOI:** 10.3390/ijms21218403

**Published:** 2020-11-09

**Authors:** Erkut Ilaslan, Renata Markosyan, Patrick Sproll, Brian J. Stevenson, Malgorzata Sajek, Marcin P. Sajek, Hasmik Hayrapetyan, Tamara Sarkisian, Ludmila Livshits, Serge Nef, Jadwiga Jaruzelska, Kamila Kusz-Zamelczyk

**Affiliations:** 1 Institute of Human Genetics, Polish Academy of Sciences, 60-479 Poznan, Poland; erkut.ilaslan@igcz.poznan.pl (E.I.); marcin.sajek@igcz.poznan.pl (M.P.S.); jadwiga.jaruzelska@igcz.poznan.pl (J.J.); 2 Endocrinology Department, “Muratsan” University Hospital, Endocrinology Clinic, Yerevan State Medical University, 0025 Yerevan, Armenia; renatamarkosyan@mail.ru; 3 Division of Endocrinology, University of Fribourg, 1700 Fribourg, Switzerland; patrick.sproll@unifr.ch; 4 SIB Swiss Institute of Bioinformatics, 1015 Lausanne, Switzerland; brian.stevenson@unil.ch; 5 Department of Human Molecular Genetics, Institute of Molecular Biology and Biotechnology, Adam Mickiewicz University, 61-614 Poznan, Poland; grete@amu.edu.pl; 6 Department of Medical Genetics, Yerevan State Medical University, 0025 Yerevan, Armenia; hasmik.hayrapetyan@cmg.am (H.H.); tamarasarkisyan@gmail.com (T.S.); 7 Center of Medical Genetics and Primary Health Care, 375010 Yerevan, Armenia; 8 Institute of Molecular Biology and Genetics, National Academy of Sciences of Ukraine, 03143 Kyiv, Ukraine; livshits@edu.imbg.org.ua; 9 Department of Genetic Medicine and Development, Faculty of Medicine, University of Geneva, CH-1211 Genève 4, Switzerland

**Keywords:** androgen insensitivity syndrome (AIS), partial androgen insensitivity syndrome (PAIS), disorder of sexual development (DSD), androgen receptor signaling, FKBP4

## Abstract

Androgen insensitivity syndrome (AIS), manifesting incomplete virilization in 46,XY individuals, is caused mostly by androgen receptor (AR) gene mutations. Therefore, a search for *AR* mutations is a routine approach in AIS diagnosis. However, some AIS patients lack *AR* mutations, which complicates the diagnosis. Here, we describe a patient suffering from partial androgen insensitivity syndrome (PAIS) and lacking *AR* mutations. The whole exome sequencing of the patient and his family members identified a heterozygous *FKBP4* gene mutation, c.956T>C (p.Leu319Pro), inherited from the mother. The gene encodes FKBP prolyl isomerase 4, a positive regulator of the AR signaling pathway. This is the first report describing a *FKBP4* gene mutation in association with a human disorder of sexual development (DSD). Importantly, the dysfunction of a homologous gene was previously reported in mice, resulting in a phenotype corresponding to PAIS. Moreover, the Leu319Pro amino acid substitution occurred in a highly conserved position of the FKBP4 region, responsible for interaction with other proteins that are crucial for the AR functional heterocomplex formation and therefore the substitution is predicted to cause the disease. We proposed the *FKBP4* gene as a candidate AIS gene and suggest screening that gene for the molecular diagnosis of AIS patients lacking *AR* gene mutations.

## 1. Introduction

Androgens govern the development of reproductive and non-reproductive pathways of the male body during the successive life periods, resulting in human sexual dimorphism. Testosterone and dihydrotestosterone are the most crucial androgens inducing male development. In the male embryo, they are responsible for the development of the Wolffian duct into the internal male sex organs and virilization of external genitalia, respectively. At the puberty period, androgens govern the growth of male internal and external genitalia as well as the growth of skeletal muscle, development of larynx, and general growth spurt. In the adult, androgens regulate spermatogenesis, muscle mass, bone metabolism as well as behavior; for a review, see [1,2,3]. Androgens act via the androgen receptor (AR) signaling pathway. Namely, they bind to the AR in the AR-Hsp90-p23 heterocomplex located within the cytoplasm of androgen-sensitive cells, cause AR activation and translocation into the nucleus, whereby AR functions as a transcription factor of specific genes; for a review, see [3,4]. The expression of these genes, in turn, stimulates cell growth, proliferation, cell cycle progression as well as secretion of specific proteins by AR-expressing cells, resulting in the development of a male phenotype; for review see [3]. The AR-mediated transcription is modulated by FKBP4 protein (also known as FKBP52), which interacts with AR [5] and its chaperon Hsp90 [6]. Thanks to the FKBP4–Hsp90 interaction, the AR receptor gains a high affinity for hormone binding and the AR-mediated transcription becomes upregulated [7,8,9,10].

Loss-of-function mutations in the *AR* gene (located on the X chromosome) cause the dysregulation of the AR signaling pathway, resulting in androgen insensitivity syndrome (AIS) in 46,XY individuals. This syndrome is characterized by abnormalities of the internal and external genitalia virilization as well as secondary sex characteristics. This occurs despite the presence of hormonally active bilateral testes producing normal levels of testosterone and anti-Müllerian hormone (AMH), for a review, see [11]. The spectrum of AIS phenotypes is broad and classified in three major categories: complete (CAIS), partial (PAIS), and mild androgen insensitivity syndrome (MAIS). CAIS patients display female phenotypic characteristics, like female external genitalia, presence of distal vagina, female breast development, and the absence of prostate and Wolffian structures. However, they lack Müllerian duct derivatives, i.e., upper vagina, Fallopian tubes, and uterus. Those phenotypic females are characterized by primary amenorrhea. The PAIS category is more variable then CAIS. The phallic structure of PAIS patients’ ranges from a penis with varying degree of diminished size and hypospadias, up to a slightly enlarged clitoris. Wolffian structures are fully or partially developed, and the prostate is typically small or impalpable. Müllerian remnants are very rare. Finally, the MAIS category is reported in men representing adolescent gynecomastia and/or suffering from infertility; for a review, see [1]. Although a majority of AIS patients carry hemizygous mutations of the *AR* gene, in some AIS individuals no *AR* gene mutations have been found. Namely, around 10% of CAIS and 60–80% of PAIS patients do not carry such mutations; for a review, see [12,13]. Studies of such patients described mutations in some other genes important for sexual development, e.g., in the hypospadias-associated *MAMLD1* gene [14] or the *NR5A1* gene, encoding steroidogenic factor 1 [15]. Nevertheless, a majority of AIS patients lacking *AR* mutations are genetically unexplained. It was hypothesized that mutations in the AR co-regulator proteins could account for the AIS phenotype [16]. According to this hypothesis it was shown that a group of AIS patients with no *AR* gene mutation revealed reduced AR transactivation ability since the mRNA level of an AR target gene was significantly lowered. This data supports the existence of cellular components, besides the AR, affecting androgen signaling during sexual differentiation and suggests their involvement in the etiology of AIS when disrupted. That phenotypic AIS subgroup characterized by a lowered expression of AR target while lacking *AR* gene mutation is called AIS type II [13]. The hypothesis that the mutations of AR co-activators could be responsible for AIS, has not been confirmed, since no such mutations have been identified so far; for a review, see [17]. Therefore, the elucidation of the molecular background of idiopathic AIS is crucial for achieving a more complete understanding of the etiology of the disease and would be precious for AIS genetic counseling.

Here, we report for the first time a potentially causative *FKBP4* gene mutation in a patient manifesting PAIS with hypospadias.

## 2. Results

### 2.1. PAIS Case Report

The chromosomal analysis of the patient showed a 46,XY karyotype without evidence of mosaicism. At the age of 10, urological examinations revealed the size of penis and testes being smaller, compared to the average at his age: penis length was 25 mm, the right testis 9 mm × 7 mm (0.33 mL), the left one 12 mm × 9 mm (0.565 mL). Pelvic ultrasound investigation did not reveal any structure of Müllerian ducts. The patient was overweight (BMI = 32.2 kg/m^2^). Later on, at the age of 13, urological examinations revealed the size of penis and testes still smaller compared to the average, namely penis length was 28 mm, the right testis size was 58 mm × 24 mm (7.28 mL), the left one was 60 mm × 21 mm (6.59 mL).

Hormonal investigations at the age of 10 revealed testosterone, anti-Müllerian hormone (AMH), estradiol, luteinizing hormone (LH), follicle-stimulating hormone (FSH), thyroid-stimulating hormone (TSH), prolactin (PRL), dehydroepiandrosterone sulfate (DHEAS), cortisol, and progesterone within the proper range, in accordance with age (Table 1). The human chorionic gonadotropin (hCG) testosterone stimulation test, performed at the age of 10 and 13, revealed a high response of testosterone, i.e., 8-fold and over 7-fold increase in testosterone concentration, respectively (Table 1). This result indicated correct testosterone biosynthesis.

Taking into consideration (1) the characteristics of the disorder of sexual development (DSD) phenotype of the patient, (2) his male karyotype, (3) the results of the hCG stimulation tests excluding defects in testosterone biosynthesis, (4) the normal AMH level excluding gonadal dysgenesis, and (5) normal LH and FSH levels excluding hypogonadotropic hypogonadism, we concluded that the patient suffered from PAIS.

### 2.2. Identification of the FKBP4:c.956T>C (p.Leu319Pro) Mutation in the Patient

**Figure 1:**
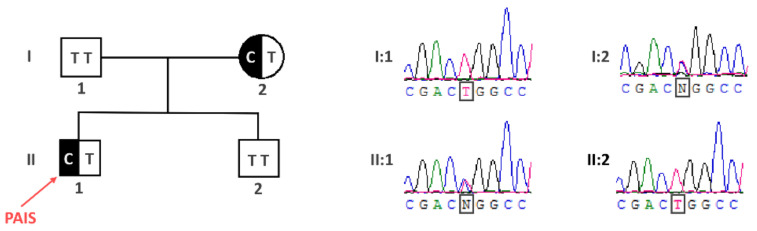
The identification of the FKBP4:c.956T>C (p.Leu319Pro) mutation in the partial androgen insensitivity syndrome (PAIS) patient. The pedigree shows the inheritance of the *FKBP4* mutation according to an autosomal dominant model, with the phenotype restricted to male individuals.

Since *AR* gene mutations are significantly less frequent in PAIS than in CAIS patients [12], we proceeded straight away with the whole exome sequencing (WES) analysis in search for the genetic background of PAIS in the patient. While the WES analysis did not reveal any *AR* gene mutation, it uncovered a heterozygous mutation in the *FKBP4* autosomal gene encoding an AR interactor [5] being a modulator of AR transcriptional activity [7,8,9,10]. The mutation was a T>C transition NM_002014.4(FKBP4_v001):c.956T>C, causing amino acid substitution NM_002014.4(FKBP4_i001):p.Leu319Pro, hereinafter referred to as FKBP4:c.956T>C (p.Leu319Pro). The patient’s mother was heterozygous for that mutation, while the father carried the wild-type alleles. We confirmed the FKBP4:c.956T>C (p.Leu319Pro) mutation status in all the family members and additionally showed a lack of mutation in the healthy brother by Sanger sequencing (Figure 1).

**Figure 2:**
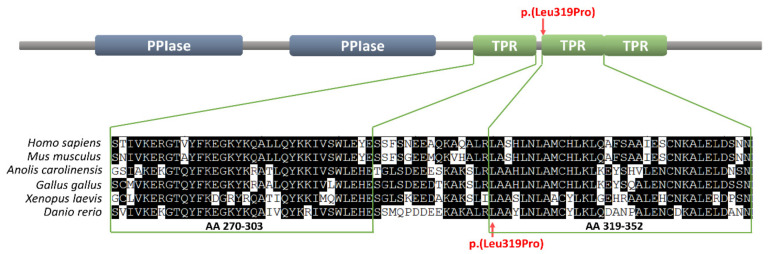
The Leu319 position within the tetratricopeptide repeat (TPR) domain of the FKBP4 protein and the conservation of that residue among vertebrates from fish to humans. The upper part represents the FKBP4 protein domains organization (PPIase = prolyl isomerase). The lower part represents the alignment of the first two human TPR repeats of human, mouse, lizard, chicken, frog, and zebrafish homologues. The FKBP4:p.Leu319Pro mutation is indicated with arrows.

Importantly, the mutation was located within one of three tetratricopeptide repeats (TPR), responsible for interaction with several proteins crucial for AR activity. Moreover, the 319 position in which the Leu319Pro substitution occurred is highly conserved among vertebrate Fkbp4 homologues (Figure 2). The FKBP4:c.956T>C (p.Leu319Pro) variant was not present in any single nucleotide polymorphism (SNP) database (gnomAD, 1000G, ExAC) and the Leu319Pro substitution in human FKBP4 protein has been predicted as disease-causing or disrupting the protein function by in silico algorithms: Meta-SNP, MutationTaster, and PolyPhen-2.

Besides the *FKBP4* gene mutation, six additional heterozygous variants in five genes (*AKR1C4*, *CYP17A1*, *FREM2*, *IL17RD*, *NKD2*) involved in sexual development were identified in the patient (among them two were identified in distinct alleles of *FREM2* gene). However, three (in *FREM2*, *IL17RD*, *NKD2* genes) out of the six variants were predicted as neutral using Meta-SNP (Table 2). For that reason, we did not further consider their influence on the patient’s phenotype. The other three variants of *AKR1C4*, *CYP17A1* or *FREM2* genes were predicted to be disease-causing (Table 2) but mutations of *CYP17A1* and *FREM2* genes caused DSD in karyotypic men only when both alleles of those genes were mutated [19,20,21], therefore the heterozygous status of *CYP17A1* and *FREM2* variants in our patient make their contribution to the DSD phenotype unlikely. However, we did not rule out the modifier effect of those variants. Besides, we cannot exclude the contribution of the *AKR1C4* variant to the DSD phenotype, all the more so that heterozygous mutations of this gene have been previously described as potential modifiers of the DSD phenotype [22].

The data of whole-exome sequencing of the described patient, his mother and his father are available from the BioProject database (ID PRJNA669263), biosample accessions: SAMN16449689, SAMN16449690, SAMN16449691.

## 3. Discussion

The number of AIS patients with no *AR* gene mutation is significant, which makes their genetic diagnosis difficult. Here, we report a patient manifesting a PAIS phenotype, lacking any *AR* gene mutation and instead carrying a novel *FKBP4* gene variant. The gene seems to be a strong candidate for PAIS, since the knockout of its homologue in the mouse caused a phenotype consistent with PAIS, including hypospadias [24]. Moreover, the function of that gene is linked to an AR signaling pathway, i.e., the FKBP4 protein interacts with AR [5] and its chaperone Hsp90 [6], leading to enhanced androgen binding and the upregulation of AR-mediated transcription [7,8,9,10]. Previously, a search for a *FKBP4* gene mutation in a group of patients with hypospadias was described but no variant associated with that condition was found [25]. The *FKBP4* variant identified in our PAIS patient carries a missense p.Leu319Pro mutation. This mutation is considered causative for several reasons. Firstly, it is located within the TPR domain, which is crucial for FKBP4-Hsp90 interaction within the AR heterocomplex, as well as for FKBP4 interaction with S100A1 and S100A2 proteins, which regulate FKBP4-Hsp90 complex formation [26]. Taking into consideration that the presence of FKBP4 in the AR heterocomplex enhances AR ligand binding, a mutation in the domain vital for FKBP4–Hsp90 interaction could impair testosterone binding by its receptor and finally causing AIS. Secondly, the Leu319Pro substitution is situated within a position, which is conserved across vertebrates (Figure 2), indicating its critical significance for FKBP4 protein function. Thirdly, the p.Leu319Pro substitution in the human FKBP4 protein has been predicted as disease-causing by several predictors of functional effects of human SNPs, and this variant is not present in any SNP database. Fourthly, the mutation in the PAIS patient described here has been inherited from its mother and was not present in the healthy brother (Figure 1). Such a pedigree may indicate an autosomal dominant model of inheritance, with the DSD phenotype restricted to karyotypic men. However, this dominant mode of inheritance is not in line with the mouse model, in which heterozygous *Fkbp4* mutant males did not display hypospadias and were fertile, in contrast to homozygous *Fkbp4* mutant animals displaying a phenotype corresponding to PAIS [24]. Still, it is possible that the gene is imprinted in humans and only the maternal allele is expressed. Alternatively, discrepancy between the human and the mouse model for dosage requirement could be taken into account, similarly to some other autosomal genes involved in sexual development. For instance, a heterozygous mutation of the *SOX8* gene, encoding a transcriptional factor closely related to SRY (the other allele being the wild-type), caused a 46,XY DSD phenotype [27]. Meanwhile, the homozygous *Sox8* knockout did not affect the sex determination process. Instead, it caused Sertoli cell function impairment in the adult, leading to infertility or decreased fertility in males [28]. Therefore, we assume that discrepancy between the phenotype of the patient carrying the *FKBP4* heterozygous variant and a lack of the abnormal phenotype in heterozygous knockout in mice does not rule out a causative effect of the *FKBP4* variant in the patient.

The six additional heterozygous variants identified in our patient in genes *AKR1C4*, *CYP17A1*, *FREM2*, *IL17RD*, *NKD2*, involved in sexual development, do not seem to be causative for the patient’s phenotype. Namely, three among these variants were predicted to be neutral (Table 2) and for that reason we did not further consider their influence on the patient’s phenotype. The other three variants of *AKR1C4*, *CYP17A1* or *FREM2* genes were predicted to be disease causing (Table 2). We consider the contribution of the *AKR1C4* variant to the DSD phenotype, as heterozygous mutations of this gene have been described as potential modifier of the DSD phenotype [22]. The mutations of *CYP17A1* and *FREM2* genes instead caused DSD in karyotypic men only when both alleles of those genes were mutated [19,20,21], therefore the heterozygous status of *CYP17A1* and *FREM2* variants in our patient make their contribution to the DSD phenotype less likely. However, we cannot rule out the modifier effect of those variants. Thus, although the described patient is a compound heterozygote of the *FREM2* gene, the variant inherited from the father was ruled out as causative due to the predicted neutral character. We assumed that the single *FREM2* variant inherited from the mother, however, predicted as disease causing is not sufficient to cause the DSD phenotype as ambiguous genitalia in karyotypic men are present in case both *FREM2* alleles are mutated [21]. Still there is possibility that the *FREM2* variant might contribute to the patient’s phenotype.

Altogether, although we do not provide direct evidence for *FKBP4* mutation causality, our findings justify placing the *FKBP4* gene within a DSD target gene panel, specifically as an AIS candidate gene. The identification of more PAIS cases carrying a *FKBP4* mutation will provide stronger confirmation for its implication in an androgen insensitivity phenotype and could contribute to the diagnosis of AIS patients lacking *AR* mutations.

## 4. Materials and Methods

### 4.1. Patient Description

A male patient, of Armenian origin, was born after full-term pregnancy (38 weeks of gestation) via vaginal delivery without complications. At birth, the patient manifested a scrotal form of hypospadias, small phallus (18 mm) with ventral chordae, bilateral cryptorchidism (testes inside the internal inguinal ring). The fourth degree of virilization was assessed according to the Prader scale. At the age of 6, the patient underwent surgery due to hypospadias, accompanied by orchiopexy. At the age of 10, a comprehensive examination, such as karyotyping, urological examinations (including testicle and pelvic ultrasound investigation), and hormonal analysis (including testosterone synthesis stimulation test) were performed. At the age of 13, urological examinations and the testosterone synthesis stimulation test were repeated. The patient expressed male psycho-sexual identity. His parents consented to endocrine and genetic studies.

### 4.2. Hormonal Analysis

At the age of 10, the patient was subjected to a complex hormonal examination. The levels of AMH, LH, FSH, TSH, PRL, DHEAS, cortisol, and progesterone were determined using the cobas e 411 instrument (Roche Diagnostics, Risch-Rotkreuz, Switzerland) and applying commercial kits (catalogue numbers: 6331076190, 11732234122, 11775863122, 11731459122, 3203093190, 3000087122, 6687733190, 7092539190 respectively (Roche Diagnostics, Risch-Rotkreuz, Switzerland). The efficiency of testosterone synthesis was examined upon 3-day testosterone stimulation with 1500 IU hCG (Pregnyl, Organon, India Ltd.) [29]. The hCG test was repeated at the age of 13.

### 4.3. DNA Isolation and Whole Exome Sequencing (WES)

Genomic DNA from the blood samples of the proband, his parents, and healthy brother was isolated by using the DNA mini kit (Qiagen, Hilden, Germany). Exome capture was performed on the proband and parents’ DNA samples by using the SureSelect Human All Exon v3 kit (Agilent Inc.^®^). Sequencing was performed on Illumina HiSeq 2000 device, and Fastq files were obtained using the Illumina CASAVA v1.8.1 software. The raw data were analyzed using our bioinformatic pipeline hosted on the Vital-IT Center of the Swiss Institute of Bioinformatics (SIB; [30]), as previously described [30]. WES data were analyzed using the VariantMaster software [31] in order to identify de novo variants, as well as variants with different Mendelian inheritance models (dominant with reduced penetrance, recessive or X-linked).

### 4.4. Sanger Sequencing

The mutation identified in the *FKBP4* gene was validated by Sanger sequencing in the patient, as compared to his parents and the healthy brother. Exon 8 of *FKBP4* gene, in which the mutation was identified, was amplified together with flanking intronic regions on DNA of the proband, his mother, father and brother, using FKBP4_E8_F:AACCTCTTGTGGCCATGTGT and FKBP4_E8_R:GTCACCAAGGGGAAGTTTCA primers. The obtained 386 bp amplicons were sequenced in both directions using the above primers, in separate reactions.

### 4.5. Bioinformatics

The data of the Genome Aggregation Database (gnomAD) [32], the 1000 genomes project (1000G) [33], and the Exome Aggregation Consortium (ExAC) Browser [34] were used to check for the variant allele frequency identified in genes involved in sexual development. The Meta-SNP integrating four algorithms (PANTHER, PhD-SNP, sorting intolerant from tolerant—SIFT and screening for non-acceptable polymorphisms—SNAP) [35], MutationTaster [36], and PolyPhen-2 [37] predictors of functional effect of SNP were used to predict the disease-causing variants identified in the genes involved in sexual development.

### 4.6. Accession Numbers 

Accession numbers of Homo sapiens mRNAs used are: *FKBP4* NM_002014.3, *AKR1C4* NM_001818, *CYP17A1* NM_000102, *FREM2* NM_207361, *IL17RD* NM_017563, *NKD2* NM_001271082. Accession numbers of vertebrate FKBP4 proteins used are: *Homo sapiens* NP_002005.1, *Mus musculus* NP_034349, *Anolis carolinensis* XP_008109533, *Gallus gallus* NP_001006250, *Xenopus laevis* NP_001084593, *Danio rerio* XP_005173673.

## Figures and Tables

**Table 1:** Values of testosterone (baseline and after human chorionic gonadotropin (hCG) stimulation), anti-Müllerian hormone (AMH), estradiol, luteinizing hormone (LH), follicle-stimulating hormone (FSH), thyroid-stimulating hormone (TSH), prolactin (PRL), dehydroepiandrosterone sulfate (DHEAS), cortisol, and progesterone of the partial androgen insensitivity syndrome (PAIS) patient.

Hormone, Unit		Age 10 Years	Age 10 Years Reference Values [18]	Age 13 Years	Age 13 Years Reference Values [18]
Total testosterone, ng/dL	Baseline after hCG	2.5	<3–10	120	18–150
		20.0		970	
AMH, ng/mL		167.4	7.4–243		
Estradiol, pg/mL		0.3	<1.0		
LH, mIU/mL		0.2	0.02–0.3		
FSH, mIU/mL		0.6	0.26–3.0		
TSH, mIU/L		3.9	0.6–6.3		
PRL, ng/mL		12.3	3–18		
DHEAS, µmol/L		24.8	13–115		
Cortisol, nmol/L		289.5	140–550		
Progesterone, nmol/L		2.9	≤3.8		

**Table 2:** List of heterozygous variants in autosomal genes involved in sexual development identified in the partial androgen insensitivity syndrome (PAIS) patient.

Gene/Protein	Associated Recessive Diseases	Related Pathway	SNP ID	Frequency (gnomAD)	DNA Change	Protein Change	Mother	Father	Meta-SNP
*FKBP4*/ Peptidyl-prolyl cis-trans isomerase FKBP4	-----	AR signaling	-	-	c.956T>C	p.Leu319Pro	Het	-	Disease
*AKR1C4*/ aldo-keto reductase family 1 member C4	*AKR1C4* may act as a modifier of 46,XY disorder of sex development due to testicular 17,20-lyase deficiency [22]	Alternative pathways of testicular androgen biosynthesis	rs533399756	0.00007946	c.704T>C	p.Leu235Pro	-	Het	Disease
*CYP17A1*/ Steroid 17-alpha-hydroxylase/17,20 lyase	46,XY disorder of sex development due to isolated 17,20-lyase deficiency [19,20]	Steroid hormone biosynthesis	rs373661758	0.00001443	c.910G>A	p.Val304Met	-	Het	Disease
*FREM2*/ FRAS1-related extracellular matrix protein 2	Fraser Syndrome 2 (characterized by ambiguous genitalia in 46,XY individuals) [21]	Extracellular matrix-receptor interaction	rs200316547	0.00022000	c.996G>T	p.Gln332His	Het	-	Disease
			rs142821775	0.00002850	c.6445A>G	p.Met2149Val	-	Het	Neutral
*IL17RD*/ Interleukin-17 receptor D	Hypogonadotropic hypogonadism 18 [23]	RET signaling	-	-	c.1164G>C	p.Glu388Asp	Het		Neutral
*NKD2*/ Protein naked cuticle homolog 2	-----	Inhibitor of WNT signaling	-	-	c.809G>A	p.Arg270Lys	-	Het	Neutral

## Abbreviations

AR	androgen receptor
AIS	androgen insensitivity syndrome
PAIS	partial androgen insensitivity syndrome
CAIS	complete androgen insensitivity syndrome
DSD	disorder of sexual development
BMI	body mass index
AMH	anti-Müllerian hormone
LH	luteinizing hormone
FSH	follicle-stimulating hormone
TSH	thyroid-stimulating hormone
PRL	prolactin
DHEAS	dehydroepiandrosterone sulfate
hCG	human chorionic gonadotropin
WES	whole exome sequencing
TPR	tetratricopeptide repeat
PPIase	prolyl isomerase
SNP	single nucleotide polymorphism
FKBP4	peptidyl-prolyl cis-trans isomerase FKBP4
AKR1C4	aldo-keto reductase family 1 member C4
CYP17A1	Steroid 17-alpha-hydroxylase/17,20 lyase
FREM2	FRAS1-related extracellular matrix protein 2
IL17RD	interleukin-17 receptor D
NKD2	rotein naked cuticle homolog 2
gnomAD	The Genome Aggregation Database
1000G	The 1000 Genomes Project
ExAC	The Exome Aggregation Consortium
SIFT	sorting intolerant from tolerant
SNAP	screening for non-acceptable polymorphisms
